# Marijuana and Cannabidiol Use Prevalence and Symptom Management Among Patients with Cancer

**DOI:** 10.1158/2767-9764.CRC-23-0233

**Published:** 2023-09-22

**Authors:** Theodore M. Brasky, Alison M. Newton, Sara Conroy, Anita Adib, Neema C. Adley, Scott A. Strassels, John L. Hays, Ziva D. Cooper, Theodore L. Wagener, Erin Stevens, Jesse J. Plascak, Jessica L. Krok-Schoen

**Affiliations:** 1 Division of Medical Oncology, The Ohio State University College of Medicine, Columbus, Ohio.; 2 Center for Biostatistics, Department of Biomedical Informatics, The Ohio State University College of Medicine, Columbus, Ohio.; 3 Division of Cancer Prevention and Control, The Ohio State University College of Medicine, Columbus, Ohio.; 4 Department of Neuroscience, The Ohio State University College of Arts and Sciences, Columbus, Ohio.; 5 Atrium Health, Charlotte, North Carolina.; 6 UCLA Center for Cannabis and Cannabinoids, Jane and Terry Semel Institute for Neuroscience and Human Behavior, University of California, Los Angeles, California.; 7 Department of Psychiatry and Biobehavioral Sciences, University of California, Los Angeles, California.; 8 Department of Anesthesiology and Perioperative Medicine, University of California, Los Angeles, California.; 9 Department of Internal Medicine, The Ohio State University College of Medicine; Columbus, Ohio.; 10 School of Health and Rehabilitation Sciences, The Ohio State University College of Medicine, Columbus, Ohio.

## Abstract

Symptoms such as pain, nausea, and anxiety are common in individuals with cancer. Treatment of these issues is often challenging. Cannabis products may be helpful in reducing the severity of these symptoms. While some studies include data on the prevalence of cannabis use among patients with cancer, detailed data remain limited, and none have reported the prevalence of cannabidiol (CBD) use in this population.

Adult patients with cancer attending eight clinics at a large, NCI-designated Comprehensive Cancer Center completed a detailed, cannabis-focused questionnaire between 2021 and 2022. Eligible participants were diagnosed with invasive cancer and treated in the past 12 months. Summary statistics were calculated to describe the sample regarding cannabis use.

Approximately 15% (*n* = 142) of consented patients (*n* = 934) reported current cannabis use (defined as use within the past 12 months). Among which, 75% reported cannabis use in the past week. Among current cannabis users, 39% (*n* = 56; 6% overall) used CBD products. Current users reported using cannabis a median of 4.5 (interquartile range: 0.6–7.0) days/week, 2.0 (1.0–3.0) times per use/day, and for 3 years (0.8–30.0). Use patterns varied by route of administration. Patients reported moderate to high relief of symptoms with cannabis use.

This study is the most detailed to date in terms of cannabis measurement and provides information about the current state of cannabis use in active cancer. Future studies should include complete assessments of cannabis product use, multiple recruitment sites, and diverse patient populations.

**Significance:**

Clinicians should be aware that patients are using cannabis products and perceive symptom relief with its use.

## Introduction

Cancer-related symptoms including pain, nausea, vomiting, and anxiety are common in individuals with cancer due to the disease and its treatment (1). Preliminary observational and experimental studies suggest that marijuana and/or cannabidiol products [(CBD); products containing <0.3% delta-9-tetrahydrocannabinol (THC)] may alleviate common cancer-related and/or treatment-related problematic symptoms including chronic pain, fatigue, nausea, neuropathy, and anorexia (2–4) and may also improve impacting mood, anxiety, appetite, and sleep disturbances among adults with cancer (5, 6).

The use of cannabis products is evolving in the United States with a changing legal landscape. Results from the 2020 National Survey on Drug Use and Health indicate that over 35 million U.S. adults over the age of 25 used marijuana (i.e., products containing ≥0.3% THC) in the past year (7), constituting a 5.9% increase from 2015. National data suggest that at least 17% of adults who used marijuana in the past year used it medically (8).

Prevalence estimates of cannabis use among adult patients with cancer in the have ranged from 8% to 25% (Table 1; refs. 9–22). However, existing research has been limited. With the exception of national studies, including nationally representative (10, 11, 19, 21, 22) and online convenience sampling schemes (14, 15), all but two (9, 12) of the remaining U.S.-based studies (13, 16, 17, 20) were performed in states that have legalized nonmedical adult marijuana use, which would likely not represent cannabis prevalence among patients with cancer in states with only medical marijuana programs. Aside from these, only two prior reports have examined cannabis use among active general oncology patients (10, 13), with the remaining focused on patients attending specific clinics or cancer survivors. In addition, measurement of cannabis product use has thus far been limited with only a few studies reporting more detailed aspects of cannabis use that likely impact health outcomes (e.g., frequency, intensity, duration, or modes of use; refs. 12, 13, 15, 20). Despite increasing availability and overlapping cannabinoids (23), no prior study has reported on the use of CBD products among patients with cancer. This has led to an underestimation of the true proportion of patients utilizing cannabis products in these studies, the contribution of concurrent CBD use in symptom management, and a lack of understanding of the magnitude of CBD use in this population.

**TABLE 1:**
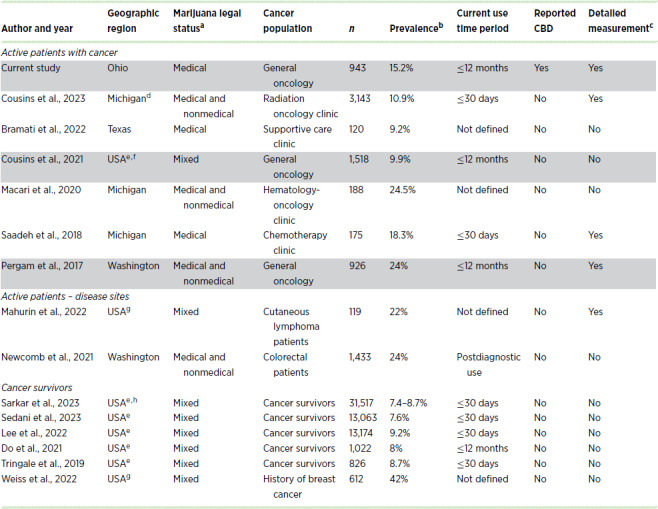
Prior studies of cannabis use among active patients with cancer and cancer survivors in the United States. Only two prior reports (highlighted) have examined cannabis prevalence among active general oncology populations

Abbreviation: USA, United States.

^a^Marijuana legal status at the time of publication (excludes CBD laws).

^b^Prevalence of current or recent cannabis use.

^c^Assessed frequency, intensity, or duration of use.

^d^Included patients with benign conditions (5%).

^e^Representative sampling using national datasets.

^f^ *n* = 1,518 recent patients with cancer; reported prevalence of cannabis use was 8.9% among 4,741 cancer survivors.

^g^Utilized online convenience sampling.

^h^Reported prevalence across 3 years.

Given the paucity of detailed information on cannabis use, there is a need to better understand the extent and patterns of cannabis use among patients with cancer under clinicians’ care. Indeed, a recent nationally representative survey of clinical oncologists reported that while 80% had discussed medical marijuana with their patients, and over 65% reported that medical marijuana was of utility for symptom management, only 30% felt sufficiently knowledgeable about medical marijuana (24). Herein, we aimed to identify the prevalence and modes of cannabis use, including marijuana and CBD products, as well as identify reasons for use and perceived effectiveness in symptom management in adult patients with cancer participating in a cross-sectional study at a large, NCI-designated Comprehensive Cancer Center (CCC).

## Materials and Methods

### Overview

From July 2021 through August 2022, adults ≥18 years of age, with a diagnosis of invasive cancer were recruited for this cross-sectional study focused on cannabis use. Participants were enrolled from eight surgical and medical oncology clinics at the Ohio State University (OSU) CCC, in Columbus, Ohio: breast, cutaneous oncology, gastrointestinal, genitourinary, gynecologic oncology, hematology, otolaryngology, and thoracic; together, these clinics encompass the most common cancers diagnosed nationally and in Ohio (25, 26). Medical marijuana was legalized in Ohio in 2016 and first became available to residents in 2018. Patients were identified via the electronic medical record by trained research assistants. Eligible patients were new or returning patients who presented to the OSUCCC with a diagnosis of invasive cancer of any anatomic site and were treated for that cancer within the past 12 months. Patients were excluded if they were diagnosed with noninvasive (i.e., *in situ*) cancer, could not speak or read English, had significant cognitive impairment or were unable to provide verbal informed consent to participate in research. The study was approved by OSU's Institutional Review Board.

### Recruitment

In partnership with OSUCCC clinical and research leaders, participants were recruited in-person during clinic visits or remotely by telephone using a nonprobability sampling scheme. For in-person recruitment, a trained research assistant entered patients’ exam rooms on a weekly rotating clinic schedule, with permission from their care team, to describe the study, answer patients’ questions, and obtain verbal informed consent. Patients consented verbally and were provided a secured iPad to complete the web-based, self-administered questionnaire after research staff left the exam room. If research staff were denied permission to approach a patient (typically due to patient distress), efforts were made to contact the patient remotely or at a subsequent appointment. For remote recruitment, study personnel called patients ≥24 hours following their in-clinic appointment, introduced themselves, explained the study, and obtained verbal consent. If the person consented to participate, the questionnaire link was provided verbally or through a secure email, depending on participant's preferences. All questionnaire responses were directly entered by participants into Research Electronic Data Capture (REDCap), a secure database (27, 28). Participants were not asked for their names, dates of birth, or addresses and neither these nor clinical data were abstracted from the medical record, to protect reporting of sensitive and/or illicit behavior. Tracking logs of patients who declined, enrolled, or were ineligible were kept to avoid recontact. All participants were offered a $10 gift card in remuneration for their time, regardless of recruitment method. Participants who completed the research questionnaire remotely were asked to click a link to a separate REDCap form to supply an address for their gift card to be mailed to ensure that participants’ questionnaire responses could not be linked to an address.

### Data Collection

A modified version of a cannabis-focused questionnaire designed and validated in patients with cancer (13) was utilized and expanded upon to include additional details on cannabis use behaviors. Patients were asked about specific products used (marijuana vs. CBD products), modes of use [i.e., inhaled (smoked or vaporized), consumed, applied to the skin, or “other” products] and weekly frequency, intensity (i.e., occasions of use per use/day), duration of use, and timing of cannabis initiation relative to patients’ cancer diagnosis. Patients were not asked about use of the antiemetic, dronabinol, a synthetic form of THC. Participants were further asked about where they obtain cannabis products, their symptoms/reasons for use, and the degree to which cannabis relieved those symptoms [scale of 1 (minimal) to 10 (major)]. Participants were also asked about their cancers, including anatomic site, spread, cancer treatment, and use of medications for cancer-related symptoms. We additionally ascertained age, gender, race, ethnicity, education level, area of residence (state and zip code), and tobacco use.

### Data Analysis

We defined current cannabis use as self-identified current use, restricted to use within the past 12 months (13). Past users were considered those who self-identified as such, and who stopped using cannabis at any time prior to participation in the study. We summarize categorical data as frequencies and proportions (%), and continuous data with medians and interquartile ranges (IQR). Selected participant characteristics are described for the sample (*n* = 943), and current cannabis use data are described for current users (*n* = 148) and stratified on mode of cannabis use. As participants can interact with cannabis products in multiple ways, some data are presented in overlapping/nonmutually exclusive categories. SAS version 9.4 was used for data management and statistical calculations.

### Data Availability

Raw data for this study were generated at the OSUCCC; derived data supporting the study's findings are available upon reasonable request of the study's corresponding author.

## Results

### Study Sample

A total of 1,692 patients were identified in the respective clinics and deemed eligible. Among them, 1,284 were able to be contacted either in-clinic or remotely. Among the 1,284 patients contacted, 1,076 (83.8%) consented into the study; 133 (12.4%) of these patients did not complete the questionnaire. Finally, we excluded 9 patients who did not provide cannabis information. The final study was comprised of 934 patients among whom 714 were consented in the clinic and 220 were consented remotely.

Selected participant characteristics are given in Table 2, leftmost column. Participants were a median of 63 years of age (IQR: 54.0–70.0). There was a slight majority (55%) of female-identifying patients, and 89% identified as non-Hispanic White. Approximately 70% of participants had attended college, and 94% were Ohio residents. Most participants (81%) were undergoing cancer treatment at the time of consent and 49% reported that their disease was locally confined. Approximately 54% of the study sample was comprised of patients who reported having lung, breast, melanoma skin, or hematologic cancers.

**TABLE 2:** Selected characteristics among 934 patients with invasive cancer, overall and stratified on current cannabis use

		Current cannabis use, *n* (%)^b^	
Characteristic	Overall, *n* (%)^a^	Yes, *n* = 142	No, *n* = 792
Age, years; median (IQR)^c^	63.0 (54.0–70.0)	56.0 (48.0–64.0)	64.0 (56.0–71.0)
Gender			
Female	513 (55.2)	82 (16.0)	431 (84.0)
Male	413 (44.2)	59 (14.3)	354 (85.7)
Prefer not to say	2 (0.2)	0 (0.0)	2 (100.0)
Race			
Asian	10 (1.1)	1 (10.0)	9 (90.0)
Black	65 (7.0)	10 (15.6)	54 (82.4)
More than one race	17 (1.8)	1 (6.7)	14 (93.3)
Other	8 (0.9)	2 (25.0)	6 (75.0)
White	829 (89.2)	125 (15.2)	700 (84.9)
Education			
≤ High school diploma	272 (29.6)	53 (19.5)	219 (80.5)
Some college	304 (33.0)	55 (18.1)	249 (81.9)
≥ College degree	344 (37.4)	32 (9.3)	312 (90.7)
State of residence			
Ohio	869 (94.1)	138 (15.9)	731 (83.1)
Border state	44 (4.8)	3 (6.8)	41 (93.2)
Non-border state	11 (1.2)	0 (0.0)	11 (100.0)
*Clinical characteristics*			
Recruiting clinic			
Breast	164 (17.6)	23 (14.0)	141 (86.0)
Cutaneous oncology	97 (10.4)	9 (9.3)	88 (90.7)
Gastrointestinal	154 (16.5)	31 (20.1)	123 (79.9)
Genitourinary	98 (10.5)	13 (20.1)	85 (86.7)
Gynecologic oncology	89 (9.5)	13 (14.6)	76 (85.4)
Hematology oncology	114 (12.2)	17 (14.9)	97 (85.1)
Otolaryngology	29 (3.1)	7 (24.1)	22 (75.9)
Thoracic	189 (20.2)	29 (15.3)	160 (84.7)
Treatment received			
Currently receiving treatment	748 (80.6)	109 (14.6)	639 (85.4)
Finished treatment	141 (15.2)	23 (16.3)	118 (83.7)
Not yet received treatment	36 (3.9)	6 (16.7)	30 (83.3)
Do not plan to receive treatment	3 (0.3)	0 (0.0)	3 (100.0)
Spread of disease			
Local	463 (49.4)	67 (14.6)	392 (85.4)
Regional	119 (12.7)	16 (13.5)	103 (86.6)
Distant	150 (16.0)	24 (16.1)	125 (83.9)
Do not know^d^	205 (21.9)	32 (15.8)	170 (84.2)
Cancer site			
Lung	180 (19.3)	28 (15.6)	152 (84.4)
Hematologic	100 (10.7)	15 (16.0)	85 (85.0)
Head and neck	24 (2.6)	4 (16.7)	20 (83.3)
Other	29 (3.1)	5 (17.2)	24 (82.8)
Multiple cancers	59 (6.3)	16 (27.1)	43 (72.9)
*Reproductive cancers*	274	35 (12.8)	239 (87.2)
Breast	153 (16.4)	21 (13.7)	132 (86.3)
Ovary	51 (5.5)	7 (13.7)	44 (86.3)
Prostate	34 (3.6)	3 (8.8)	31 (91.2)
Uterus/endometrium	27 (2.9)	1 (3.7)	26 (96.3)
Other reproductive cancer	9 (1.0)	3 (33.3)	6 (66.7)
*Gastrointestinal cancers*	131	25 (19.1)	106 (80.1)
Colorectum	53 (5.7)	12 (22.6)	41 (77.4)
Pancreas	32 (3.4)	9 (28.1)	23 (71.9)
Esophagus	16 (1.7)	1 (6.3)	15 (93.8)
Other gastrointestinal cancer	30 (3.2)	3 (10.0)	27 (90.0)
*Urinary cancers*	52	6 (11.5)	46 (88.5)
Bladder	26 (2.8)	3 (11.5)	23 (88.5)
Kidney	26 (2.8)	3 (11.5)	23 (88.5)
*Skin cancers*	85	8 (9.4)	77 (90.6)
Melanoma	70 (7.5)	6 (8.6)	64 (91.4)
Non-Melanoma	15 (1.6)	2 (13.3)	13 (86.7)

^a^Valid (i.e., non-missing) column percentages.

^b^Valid row percentages.

^c^Restricted to *n* = 677 who provided age data.

^d^Participants affirmed their cancer has spread but were unsure of the extent of spread.

### Cannabis Use

Current cannabis use (including any CBD or marijuana-based product) was reported by 15% (*n* = 142/934) of study participants and overall, 14% (*n* = 131/934) used cannabis in the past 30 days (Table 2). Current cannabis users were younger than nonusers, and the prevalence of use was highest among those with lower education and among participants who had other reproductive cancers (33%; *n* = 7 cervical and *n* = 2 testicular cancers), pancreatic cancer (28%), multiple cancers (27%), or colorectal cancer (23%). Participants with uterine (4%), esophageal (6%), or prostate cancer (9%) had the lowest prevalence of use. There was no clear difference in cannabis use prevalence by spread of disease or stage of treatment.

Cannabis use characteristics among current users stratified on modes of use are depicted in Table 3. Among 142 current cannabis users, 86 inhaled cannabis, 94 consumed it, and 13 applied cannabis products to their skin (Table 3 column headers), with 35% reporting >1 mode of use. Among all current users, 71% did not have a medical marijuana prescription, and 34% initiated cannabis use after their cancer diagnosis. Most patients (75%) currently using cannabis had used a cannabis product in the past week. Approximately 9% exclusively used CBD products, 37% exclusively used marijuana products, and 31% used both CBD and marijuana products. A quarter of current cannabis users (23%) were unsure of the type of product they used. The proportion of exclusive marijuana use was highest for inhaled products (69%) and lowest for topically applied products (8%). Among the entire study population (*n* = 934), CBD use prevalence was 6%, with 1% exclusive use, and marijuana use prevalence was 10%, with 6% exclusive use.

**TABLE 3:** Patterns of current cannabis product use (*n* = 142)

	**Current cannabis users, *n* (%)** ^a^ ^,^ ^b^			
	**Overall, *n* = 142**	**Inhaled, *n* = 86**	**Consumed, *n* = 94**	**Applied, *n* = 13**
Mode of use^b^^,^^c^				
Poly-use	49 (35.0)			
Inhaled	42 (30.0)			
Consumed	46 (32.9)			
Applied	3 (2.1)			
Medical marijuana prescription				
No	101 (71.1)			
Yes	41 (28.9)			
Initiation of cannabis use				
Before cancer diagnosis	91 (66.4)			
After cancer diagnosis	46 (33.6)			
Time since last cannabis use (days)				
Today	40 (28.2)			
1–7	67 (47.2)			
8–30	24 (16.9)			
>30	11 (7.7)			
Type of product used^d^				
Cannabidiol (CBD) only	12 (8.5)	2 (2.4)	8 (8.6)	6 (46.2)
Marijuana only	53 (37.3)	59 (69.4)	40 (43.0)	1 (7.7)
Both	44 (31.0)	16 (18.8)	34 (36.6)	5 (38.5)
Do not know	33 (23.2)	8 (9.4)	11 (11.8)	1 (7.7)
Frequency of cannabis use (days/week)				
<1	36 (25.4)	17 (20.0)	32 (34.8)	8 (61.5)
1–6	46 (32.3)	27 (31.8)	33 (35.9)	2 (15.4)
7	60 (42.3)	41 (48.2)	27 (29.4)	3 (23.1)
Median (IQR)	4.5 (0.6–7.0)	5.5 (2.0–7.0)	3.5 (0.6–7.0)	0.6 (0.6–3.5)
Intensity of cannabis use (occasions per use/day)				
1	62 (44.3)	23 (27.0)	53 (60.2)	1 (100.0)
2	28 (20.0)	22 (25.9)	16 (18.2)	0 (0)
3	30 (21.4)	18 (21.2)	14 (15.9)	0 (0)
≥4	20 (14.3)	22 (25.9)	5 (5.7)	0 (0)
Median (IQR)	2.0 (1.0–3.0)	2.0 (1.0–3.3)	1.0 (1.0–2.0)	1.0 (1.0–1.0)
Duration of current use (years)				
<1	35 (25.3)	10 (12.2)	37 (42.5)	3 (23.1)
1–2	24 (18.1)	8 (9.8)	27 (31.0)	7 (53.9)
>2	74 (55.6)	64 (78.1)	23 (26.4)	3 (23.1)
Median (IQR)	3.0 (0.8–30.0)	20.0 (3.0–40.0)	1.0 (0.3–3.0)	2.0 (1.0–2.0)

^a^Valid column percentages reported.

^b^Excludes frequency, intensity, duration, and product type data for *n* = 5 (*n* = 2 exclusive users) who use “Other” cannabis products.

^c^Mutually exclusive categories.

^d^Cannabidiol products defined as cannabis products containing <0.3% THC; marijuana products defined as cannabis products containing ≥0.3% THC.

The median weekly frequency of cannabis use across all modes of administration was 4.5 days (IQR: 0.6–7.0); frequency of use for inhalation was higher (5.5, IQR: 2.0–7.0) than frequencies for consumed (3.5, IQR: 0.6–7.0) and topically applied products (0.6, IQR: 0.6–3.5). On days patients reported using cannabis, the median was 2 times/day (IQR: 1.0–3.0), with less intense use for consumed and applied products. Cannabis use was more frequent among those with a medical marijuana prescription and those who initiated cannabis use prior to their diagnosis (Supplementary Table S1). The median time since initiation of current cannabis use was 3 years (IQR: 0.8–30.0); however, this varied widely depending on the mode of administration, with inhaled products having been used for a median of 20 years (IQR: 3.0–40.0) and consumed or topical products used for a median of 1 to 2 years, respectively.

**FIGURE 1:**
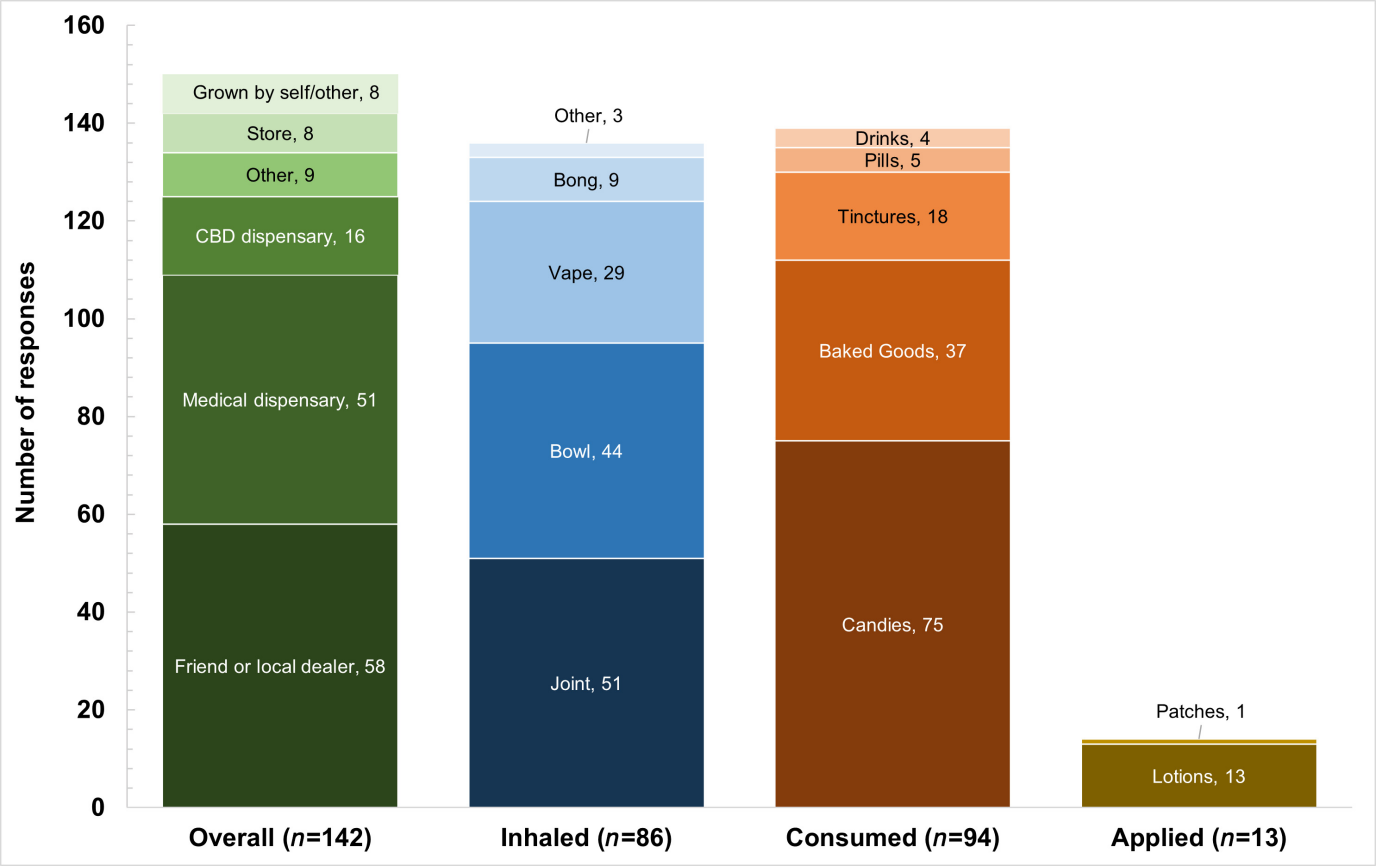
Methods of obtaining cannabis products and modes of current cannabis product use among 142 patients with cancer. Participants took cannabis in multiple ways and category sample sizes do not sum to 142. Response options for how cannabis is obtained or details on modes of use are also nonmutually exclusive and do not sum to category totals. Category “other” (*n* = 5 responses) is not pictured above.

Figure 1 illustrates how cannabis products were obtained and the frequency of responses for how products were used, stratified on mode of use. Among current users, the top means of obtaining cannabis products were from a friend or local dealer (39%), or a medical dispensary (34%). Joints (38%) and bowls (32%) were the most popular method of inhalation, whereas candies (54%) and baked goods (27%) were the most popular ingested products. Cannabis-based lotions were the most popular method of topical application (93%).

### Reasons for Cannabis Use

**FIGURE 2:**
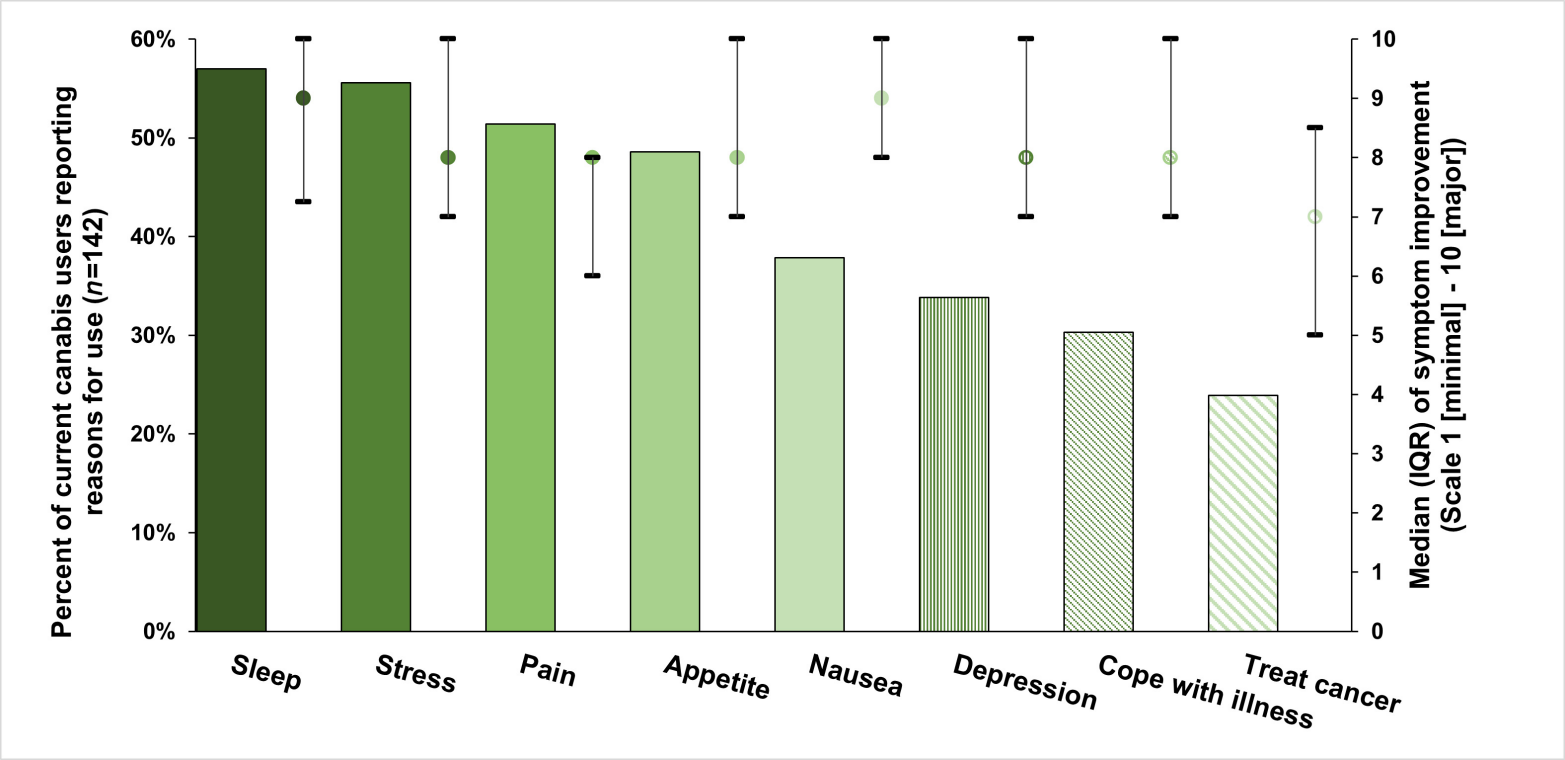
Proportion of 142 current cannabis users reporting reasons for cannabis use (left ordinate; bars), and medians and quartile ranges (right ordinate; dots and lines) of the degree to which participants reported symptom relief from cannabis (scale of 1 to 10). Reasons for use are nonmutually exclusive and data do not sum to 100%. Not shown: 28% reported use of cannabis products for enjoyment/recreation. Different than symptom-related reasons for use, participants were not asked a follow-up question to rank enjoyment/recreation.

Reasons for cannabis use among the 142 current cannabis users are presented in Fig. 2. The top five reasons for use were sleep (57%), stress (56%), pain (51%), appetite (49%), and nausea (38%). Twenty-eight percent of current users reported cannabis use for recreation, although only 2% did so exclusively. Participants reported that cannabis provided more than moderate relief of their symptoms. On a scale of 1 (minimal relief) to 10 (major relief), the highest median (IQR) reported relief was for sleep [9 (IQR: 7.3–10)] and nausea [9 (IQR: 8–10)]. Patients reported the least relief regarding treatment of their cancer [median (IQR): 7 (5–7.5)]. Median values for relief of stress, pain, anxiety, depression, and coping were all 8.

## Discussion

This study examined the frequency, intensity, routes of administration, duration, timing, and types of cannabis use in adults with invasive cancer at a comprehensive cancer center in Columbus, Ohio. To our knowledge, this study is the first to distinguish between marijuana and CBD products among patients with cancer.

We found that 15.2% of the patient population reported cannabis-product use overall, which is consistent with previously reported rates of 9% to 25% (Supplementary Table S1) among active patients (9–13, 17, 19, 20). Notably, our findings differ from nationally representative datasets, which estimate the prevalence of use to be below 10% (10, 11, 19, 21). These lower estimates may reflect differences in the legal status of cannabis across states as well as inclusion of individuals who reported a past history of cancer (11, 19, 21). Only two prior studies have examined the magnitude of cannabis use among active general oncology patients (10, 13), whereas most prior reports are limited to individual treatment clinics (e.g., supportive care, radiation oncology, etc.) or national studies typically among cancer survivors (Table 1). Our reported prevalence of 15.2% current cannabis use contrasts with several prior reports from Michigan, which is the only other midwestern state for which data are available. Saadeh and colleagues (12) reported 18.3% current cannabis use among 175 patients attending a chemotherapy clinic at a community cancer center. At the time the research was conducted, nonmedical cannabis use had not yet been legalized, making the legal context most like the current study, but the underlying population of patients receiving chemotherapy is markedly different. In two subsequent studies from Michigan, completed after nonmedical cannabis use was legalized, Macari and colleagues (17) and Cousins and colleagues (20) report prevalence rates of 24.5% and 10.9% among patients in hematology-oncology and radiation oncology clinics, respectively. We posit that the differences between the current study and the three prior reports from Michigan are likely due to several factors, including differences in the cancer populations under study, as well as differences in how cannabis use was defined and measured. Prevalence reports from the few remaining single institute general oncology studies (9, 13) may have been influenced by eligibility criteria (e.g., specific clinics), cannabis measurement, or nonmedical cannabis legal status and availability.

How authors define current (active, recent) cannabis use plays a role in reported prevalence rates and, in conjunction with other factors noted above, likely helps explain differences across studies (Table 1). We defined current cannabis use herein as self-identified current users who last used cannabis within the past 12 months, similar to Pergam and colleagues (13). In contrast, some nationally representative studies reported on past-year use irrespective of self-identification of current use status, thereby including as “current use” patients with cancer and survivors who may have stopped (either on their own or at the behest of their physician; refs. 10, 11). Yet others have reported on cannabis use in the past 30 days (12, 19–22, 29). We report 15.2% and 14% cannabis prevalence in the past year and past 30 days, respectively, among patients who self-identified as current users. If we had instead included patients who reported that they stopped using cannabis in our definition of “current users,” the prevalence would have been 22.6% and 15.1% for use in the past year and past 30 days, respectively.

Some investigators have reported cannabis prevalence among patients with cancers of specific sites or site groupings (refs. 9, 13–16; Table 1). Like our findings, patients with colorectal and other gastrointestinal cancers had among the highest cannabis use prevalence (9, 13, 16). In contrast, Mahurin and colleagues (15) and Weiss and colleagues (14) reported higher prevalence proportions among patients with cutaneous lymphoma (22%) and breast cancer (42%) than we observed for patients diagnosed with hematologic (11%) and breast cancers (16%), respectively. However, these studies utilized web-based sampling, without verifying cancer status, recency, or residential cannabis laws which may have artificially inflated reported prevalence rates (18). In Ohio, where this study took place, nonmedical marijuana use remains illegal, and fewer than 30% of current cannabis users reported attaining cannabis-based products through a medical cannabis prescription. If legislation to legalize marijuana were to become more common, as appears to be the case (7), the prevalence of marijuana use among patients with cancers would be expected to further increase.

To our knowledge, this study is the first to examine use of the full complement of cannabis-based products, including marijuana and CBD products, among patients with cancer. Among all patients, the prevalence of CBD use was 6% (40% among current cannabis users). CBD has been observed to have anti-inflammatory and analgesic properties (30, 31) and contains overlapping cannabinoids with marijuana products. Given this, its growing popularity and easy accessibility, and its evolving legal status at state and federal levels, investigators should strongly consider measuring all cannabis-based products in patients with cancer.

The most common methods of cannabis use among current users in our sample were poly-use (inhalation and ingestion, 35%), closely followed by ingestion (33%) and inhalation (30%). This finding corresponds with Pergam and colleagues, that found cancer patients’ modes of marijuana use were inhalation and ingestion (40%), ingestion only (30%), and inhalation only (29%). Oral ingestion is rapidly becoming a prominent method of cannabis use, and there is a proliferation of cannabis-infused candy, beverages, and other food products (32). A potential reason for the multiple methods of administration may be due to differences in the pharmacokinetics and pharmacodynamics of respective cannabinoids, THC and CBD. For example, ingestion of cannabis products leads to delayed, but prolonged peak plasma levels of respective cannabinoids relative to inhalation (33). These differences in absorption correspond to differences in cannabis’ effects. Better understanding patients’ decision-making process regarding different methods of use is an area for future research.

Patients with cancer may experience a myriad of negative physical and psychologic symptoms related to their cancer and its treatment (1, 34, 35). We found that the top reported reasons for cannabis use were to help patients sleep, deal with stress, reduce pain, and improve appetite/nausea. This is consistent with prior reports among patients with cancer in comprehensive cancer centers (13), community centers (12), and within nationally representative datasets (11). We also found moderate-high perceived effectiveness of cannabis-product use for symptom management, corresponding with the current literature (13, 36). Longitudinal observational studies of patients who use medical marijuana for symptom management during their cancer treatment reported significantly lower levels of depression (6, 37, 38), reduced anxiety (6, 37–39), and discontinued anxiolytic (i.e., anti-anxiety) medications (6). There are clinical trial data on cannabis-based medicines for chronic pain in patients with cancer (38, 40, 41); however, data focusing on sleep disorders and emotional distress in patients with cancer are very limited (36, 41). Trial data support a beneficial effect of cannabinoids and cannabis-based products for chronic pain (specifically THC or THC-like compounds), although findings are complicated by pain type and compound used. Our finding adds to the current literature of cannabis use and unresolved problematic symptoms (pain, anxiety, reduced appetite) among patients with cancer (4, 38, 42). More research is needed as there is limited evidence that supports cannabis for the symptom sequelae experienced by patients with cancer (41). The prevalence of cannabis use coupled with the lack of established evidence and varying legality is a challenge to clinicians in their clinical assessments, recommendations, treatments, and information provision.

The current study has several strengths, including its large sample size, clinical chart review for eligibility confirmation, and detailed examination of cannabis product use, including CBD products, among a sample of patients with cancer. Furthermore, it is only the third study conducted within a state without legalized nonmedical marijuana. This study also has several limitations. Chief among them, it is possible that patients chose to participate in the research based upon their use of cannabis products, potentially leading to an overrepresentation of cannabis use in this population. That cannabis prevalence in this study was within the range of most prior reports argues against significant bias but does not dismiss the concern. This study's generalizability may be limited as 89% of the sample was non-Hispanic White (reflective of the OSUCCC's patient pool) and data were collected from a single academic cancer center. Marijuana remains an illicit drug on the federal level and is only legal for medicinal purposes in Ohio, which could lead to underreporting of cannabis use as well as patient reluctance toward study participation and subsequent sampling bias. However, our consent rate was over 80% of patients contacted. In addition, we did not query participants on marijuana doses or marijuana to CBD ratios. As such, any cannabis product containing ≥0.03% THC was considered a marijuana product regardless of CBD content and will have contributed to measurement error. This study is focused on current cannabis use behaviors in a sample of patients with cancer where the majority used cannabis prior to diagnosis. Therefore, reasons for current use cannot be distinguished from reasons for past use. Likely a reflection of the intended anonymity of the study, 28% of patients did not provide their age. This study of 934 patients with cancer was limited by 142 current cannabis users. Therefore, analyses stratified on specific characteristics among current users were limited, and some analyses (e.g., symptom relief) were limited to overall cannabis use regardless of form, mode of administration, or timing of cannabis initiation. Finally, the questionnaire was in English and not translated to other languages, which could have excluded members of minoritized racial and ethnic groups.

## Conclusions

This cross-sectional study found 15% prevalence of cannabis use among adult patients with cancer treated at an Ohio comprehensive cancer center. The reasons for cannabis use focused on symptom management (pain, anxiety, sleep disturbance) and participants reported cannabis was effective for symptom relief. This study adds to the growing literature on cannabis use in cancer patient populations. Clinicians should be aware that a substantial percentage of patients with cancer are using cannabis products and report experiencing symptom relief with its use. With an evolving legislative landscape that is likely to grow more permissive toward cannabis use, the prevalence of cannabis use among patients with cancer can be anticipated to increase in the future. Longitudinal studies to better understand trajectories of cannabis use and its association with symptom burden and management strategies among patients with cancer, as well as qualitative approaches to better understand the experiences and decision-making of patients with cancer regarding cannabis use are needed.

## Supplementary Material

Table S1 Patterns of current cannabis product use by medical marijuana prescription status and timing of cannabis initiation.

Supplementary Data Research Questionnaire
