# Supplementary material for: Marijuana and Cannabidiol Use Prevalence and Symptom Management Among Patients with Cancer
Source: Cancer Res Commun. 2023 Sep 22;3(9):1917–26. doi: 10.1158/2767-9764.CRC-23-0233 (PMC10515742; doi:10.1158/2767-9764.CRC-23-0233)
Supplement: Table S1 — Patterns of current cannabis product use by medical marijuana prescription status and timing of cannabis initiation. [file crc-23-0233-s01.docx]

| **Table S1.** Patterns of current cannabis product use by medical marijuana prescription status and timing of cannabis initiation. | | | | |
| --- | --- | --- | --- | --- |
|  | **Medical marijuana prescription,**  **median (IQR)** | | **Cannabis initiation,**  **median (IQR)** | |
|  | **Yes, n=41** | **No, n=101** | **Before cancer diagnosis, n=91** | **After cancer diagnosis, n=46** |
| Frequency of cannabis use (days/week) | 7.0 (3.5-7.0) | 3.5 (0.6-7.0) | 5.5 (2.0-7.0) | 3.5 (0.6-7.0) |
|  |  |  |  |  |
| Intensity of cannabis use (occasions per use/day) | 2.0 (1.0-3.0) | 2.0 (1.0-3.0) | 2.0 (1.0-3.0) | 1.0 (1.0-3.0) |
|  |  |  |  |  |
| Duration of current use (years) | 2.0 (0.5-10.0) | 10.0 (1.0-37.3) | 20.0 (3.0-40.0) | 0.5 (0.3-1.1) |
