# Supplementary material for: Marijuana and Cannabidiol Use Prevalence and Symptom Management Among Patients with Cancer
Source: Cancer Res Commun. 2023 Sep 22;3(9):1917–26. doi: 10.1158/2767-9764.CRC-23-0233 (PMC10515742; doi:10.1158/2767-9764.CRC-23-0233)
Supplement: Supplementary Data — Research Questionnaire [file crc-23-0233-s02.docx]

***IN CLINIC RECRUITMENT***

*[Before Research Coordinators enter patients’ exam rooms they will select the cancer clinic they are in to inform question 1 pertaining to cancer site]*

**Clinic the patient is visiting for their appointment:** *[After a Research Assistant selects the cancer clinic, a drop down box will list specific examples of cancers for participants to select their specific type of cancer in question 1]*

🞏 Breast Clinic

🞏 Dermatology Clinic

🞏 Gastroenterology Clinic

🞏 Genitourinary Clinic

🞏 Gynecologic-Oncology Clinic

🞏 Hematology-Oncology Clinic

🞏 Otolaryngology Clinic

🞏 Thoracic Clinic

**Introduction:** Thank you for participating in this anonymous survey. The information you provide is completely confidential and will not be linked to you. Before you start we want to give you definitions of words that we will be using in the survey to help you understand the questions.

**Cannabis** is a plant, and there are two main types. The first type is called **hemp** which contains **CBD** (cannabidiol). **CBD** does not have any psychoactive properties and does not get you high. The second type of cannabis is called **marijuana** which contains both **CBD** and **THC** (tetrahydrocannabinol). **THC** is the chemical that gives you a high. If you have any questions while you are taking the survey please ask the researcher for clarification.

***REMOTE RECRUTMENT***

WHO: Patients receiving care at Ohio State University Comprehensive Cancer Center treated for cancer in the past year.

WHAT: We are studying cannabis use among cancer patients and are interested in your experiences. The study consists of a brief anonymous survey. We will ask some basic information like your age, gender, race, county/zip code you reside but we will NOT record identifying information like your name, address, phone number, medical information, date of birth or any information that could identify you. You will be given a $10 Walmart gift card for completing the survey.

WHEN: The brief anonymous survey will be completed electronically and will take between 5-15 minutes of your time.

WHY: The information you provide will increase our understanding of the role that cannabis use plays in the health and treatment of cancer patients. This knowledge may be significant for physicians, cancer patients, and regulatory agencies.

OTHER IMPORTANT INFORMATION:
We are not collecting identifying information and cannot link your name to anything you answer. Since this is an anonymous survey, there is no risk of a breach of confidentiality and no expected risks of participation. Your anonymous survey responses may be used or shared with other researchers without your additional informed consent. Participation is voluntary. If you decide not to participate, there will be no penalty or loss of benefits to which you are otherwise entitled. You can decline to answer any question, as well as stop participating at any time, without any penalty or loss of benefits to which you are otherwise entitled.

Please click "next" to continue if you agree to participate and complete the survey.

**Introduction:** Thank you for participating in this anonymous survey. The information you provide is completely confidential and will not be linked to you. Before you start we want to give you definitions of words that we will be using in the survey to help you understand the questions.

**Cannabis** is a plant, and there are two main types. The first type is called **hemp** which contains **CBD** (cannabidiol). **CBD** does not have any psychoactive properties and does not get you high. The second type of cannabis is called **marijuana** which contains both **CBD** and **THC** (tetrahydrocannabinol). **THC** is the chemical that gives you a high. If you have any questions while you are taking the survey please ask the researcher for clarification.

**Which clinic have you recently visited at The James (OSU) for your cancer appointment?**

**If more than one apply, please identify the most recent clinic you have visited.**

🞏 Breast Clinic

🞏 Dermatology Clinic

🞏 Gastroenterology Clinic

🞏 Genitourinary Clinic

🞏 Gynecologic-Oncology Clinic

🞏 Hematology-Oncology Clinic

🞏 Otolaryngology Clinic

🞏 Thoracic Clinic

**ALL PARTICIPANTS**

**1. What type of cancer are you visiting the cancer center for?** *[If “other” is selected for any cancer include cancers under the “other cancers” section below. If respondent selects “other cancer not listed” provide text box for write in response].*

🞏 If Gastroenterology Clinic Gastrointestinal Clinic Selected:

🞏Anal 🞏Colon 🞏Esophageal 🞏Liver 🞏Rectal 🞏Stomach 🞏Gallbladder 🞏Pancreas 🞏Other

🞏 Hematology-Oncology Clinic Selected:

🞏Leukemia 🞏Lymphoma 🞏Multiple Myeloma 🞏Other

🞏 Gynecologic-Oncology Selected:

🞏Cervical 🞏Endometrial/Uterine 🞏Vaginal 🞏Ovarian 🞏Other

🞏 Thoracic Clinic Selected:

🞏Lung 🞏Esophageal 🞏Mesothelioma 🞏Other

🞏 Otolaryngology Clinic Selected:

🞏Oral (lip, tongue, palate) 🞏Tonsil 🞏Nasal 🞏Thyroid 🞏Laryngeal 🞏Pharyngeal 🞏Parotid or salivary gland 🞏Other

🞏 Genitourinary Clinic Selected:

🞏Prostate 🞏Bladder 🞏Kidney 🞏Testicular 🞏Penile 🞏Adrenal 🞏Ureter 🞏Other

🞏 Dermatology Clinic Selected:

🞏Melanoma (including melanoma of the skin, eye, or other parts of the body) 🞏Non-Melanoma skin 🞏Other

🞏 Breast Clinic Selected:

🞏 Invasive Ductal or Lobular Carcinoma 🞏Inflammatory Breast Cancer 🞏Unknown Breast Cancer 🞏Other

*[If ‘Other’ is selected, include additional cancer site options below as well as cancers listed above (not a complete list)]*

**Other cancers:**

Adrenal

Anal

Appendicular

Bladder

Brain

Breast—Invasive Ductal or Lobular Carcinoma

Breast—Inflammatory Breast Cancer

Breast—Unknown Breast Cancer

Cervical

Colon

Endometrial/Uterine

Esophageal

Gallbladder

Kidney

Laryngeal

Leukemia

Liver

Lung

Lymphoma

Melanoma skin

Mesothelioma

Multiple Myeloma

Myelodysplastic Syndrome

Nasal

Neuroendocrine

Non-Melanoma skin

Oral (lip, tongue, palate)

Osteosarcoma (bone)

Ovarian

Pancreas

Parathyroid

Parotid or salivary gland

Penile

Pharyngeal

Prostate

Rectal

Sarcoma (not bone)

Stomach

Testicular

Tonsil

Thyroid

Ureter

Vaginal

🞏 Other cancer not listed: ______________________________

**2. WHERE have you received information about cannabis use and cancer?** (Check all that apply)

🞏 I haven’t received this information 🞏 Another cancer patient

🞏 From a cancer doctor or nurse 🞏 Medicinal marijuana store

🞏 A doctor or nurse outside of my cancer team 🞏 Pamphlet or handout

🞏 Nutritionist 🞏 Newspaper / magazine article

🞏 Naturopath / herbalist 🞏 TV / radio advertisement

🞏 Friend / family member 🞏 Social media (Facebook, Twitter, etc.)

🞏 Other (describe):___________________________________ 🞏 Websites or blogs

**3. Are you interested in learning more about cannabis use and cancer?** (Select one)

1 -------- 2 -------- 3 -------- 4 -------- 5 ---------- 6 -------- 7 -------- 8 -------- 9 -------- 10

Not interested Somewhat interested Very interested

**4. Where WOULD you be most likely go if you wanted to learn more about cannabis use and cancer?**

(Check all that apply)

🞏 I don’t want to learn more 🞏 another cancer patient

🞏 From a cancer doctor or nurse 🞏 Medicinal marijuana store

🞏 A doctor or nurse outside of my cancer team 🞏 Pamphlet or handout

🞏 Nutritionist 🞏 Newspaper / magazine article

🞏 Naturopath / herbalist 🞏 TV / radio advertisement

🞏 Friend / family member 🞏 Social media (Facebook, Twitter, etc.)

🞏 Other (describe):___________________________________ 🞏 Websites or blogs

**5. Has your doctor or another healthcare provider recommended that you use cannabis during your cancer treatment?** 🞏 YES *[Continue to 5a]*  🞏 NO *[Go to 6]*

**5a. Which type of healthcare provider?** (Check all that apply)

🞏 A cancer doctor 🞏 A pharmacist at the cancer center

🞏 A doctor in another clinic / hospital 🞏 A pharmacist in another clinic / hospital

🞏 A cancer nurse 🞏 A nutritionist at the cancer center

🞏 A nurse in another clinic / hospital 🞏 A nutritionist at another clinic / hospital

🞏 A naturopath / herbalist

🞏 Other healthcare provider (describe): ________________________________

**6. Marijuana is a form of cannabis that contains THC, an ingredient that causes euphoria or a “high”. Does the legalization of medical marijuana in Ohio make you more likely to use marijuana?** (Select one)

1 -------- 2 -------- 3 -------- 4 -------- 5 ---------- 6 -------- 7 -------- 8 -------- 9 -------- 10

No change Somewhat more likely Much more likely

**7. Have you ever used cannabis in any form?**

🞏 YES  *[Continue to 8]* 🞏 NO *[Go to question 28]*

**8.** **Are you currently using cannabis?**

🞏 YES *[Continue to 9]* 🞏 NO *[Go to question 8a-b]*

**8a. If you are NO longer using cannabis, when did you STOP?**

🞏 Before my cancer diagnosis 🞏 After my cancer diagnosis

**8b. Why did you stop using cannabis?** (Check all that apply)

🞏 I wanted to stop

🞏 My family wanted me to stop

🞏 My primary care doctor told me to stop

🞏 I only experimented in the past

🞏 I was concerned about the dangers of cannabis

🞏 I didn’t want cannabis to affect my judgment/thinking

🞏 I stopped for other reasons

*[If respondent selected ‘after diagnosis’ in 8a, additionally include the following response options]*

🞏 I was worried cannabis was related to my cancer diagnosis

🞏 My cancer doctor told me to stop

**9. When did you START using cannabis?**

🞏 BEFORE my cancer diagnosis 🞏 AFTER my cancer diagnosis

**10. How long ago did you first use cannabis?** *[Direct respondent to select 0 months if began use in the past 4 weeks]*

|__|__| years *and/or* |__|__| months

**11. When was the last time you used cannabis?** (Select one)

🞏 Today

🞏 Within the past week

🞏 Within the past month

🞏 Within the last 6 months

🞏 Within the past year

🞏 Over a year ago

**12. Thinking about any cannabis products you use, how often do you use any cannabis product OVERALL?** (Select one)

| 🞏 | 🞏 | 🞏 | 🞏 | 🞏 | 🞏 | 🞏 | 🞏 |
| --- | --- | --- | --- | --- | --- | --- | --- |
| Less than once per month | 1 day per month | 2-3 days per month | 1 day per week | 2 days per week | 3-4 days per week | 5-6 days per week | Daily |

**13. On days that you use any cannabis product, how many times per day do you use it?** [*DROP DOWN for respondents to choose the specific number of times they use cannabis per day]*

|__|__|

**14. Does your cancer doctor know you are using cannabis products?** (Select one)

🞏 None of my healthcare providers know

🞏 I have not told my cancer doctor/team, but another healthcare provider knows

🞏 My cancer doctor/team know

**15. Where do you get cannabis?** (Check all that apply)

🞏 I grow it myself 🞏 Someone grows it for me as part of medical co-op

🞏 I get it from a friend or a local dealer 🞏 I get it from a medical dispensary with a prescription

🞏 I get it from a non-medical CBD dispensary without a prescription

🞏 I get it from a store (online or in person)

🞏 Other (describe):____________________________________________________________________

**16. Are you getting medical marijuana prescribed by one of your doctors?**

🞏 No, I don’t have a medical marijuana prescription

🞏 Yes - from one of my cancer providers

🞏 Yes - from another doctor/provider

**17. How do you use/take cannabis?** (Check all that apply)

🞏 I inhale or smoke cannabis (e.g., bud or flower, vaporizer, water pipe, etc.) *[Continue to 18a-e]*

🞏 I eat or drink cannabis products (e.g., foods, edibles, tinctures, oils, or capsules, etc.) *[Continue to 19a-e]*

🞏 I apply cannabis to my skin (e.g., patches, creams, balms, ointments, etc.) *[Continue to 20a-e]*

🞏 Other (please tell us the type of the product you use, e.g., wax, suppositories, etc.):___ *[Continue to 21a-e]*

**18a. When you inhale or smoke cannabis, what method do you use?** (Check all that apply)

🞏 I use rolled cannabis cigarettes (“joint”)

🞏 I use a vaporizer (“vape”) or electronic-cigarette device

🞏 I use a water pipe or hookah (“bong”)

🞏 I use a pipe (“bowl”)

🞏 Other (describe): ________________________________

**18b. The State of Ohio considers "CBD products" to contain either no or almost no (not more than 0.3%) THC, the ingredient in cannabis that can cause a euphoria or a "high". Some CBD products have no THC at all. Products with higher amounts of THC are considered to be "marijuana products".**

**What type of cannabis product do you inhale or smoke? (Select one)**

🞏 No, I only inhale/smoke THC-free (CBD) cannabis products

🞏 Yes, I only inhale/smoke cannabis products that contain THC

🞏 I inhale/smoke both products that contain THC and some that do not (CBD)

🞏 I don’t know

**18c. How often do you inhale or smoke cannabis?** (Select one)

| 🞏 | 🞏 | 🞏 | 🞏 | 🞏 | 🞏 | 🞏 | 🞏 |
| --- | --- | --- | --- | --- | --- | --- | --- |
| Less than once per month | 1 day per month | 2-3 days per month | 1 day per week | 2 days per week | 3-4 days per week | 5-6 days per week | Daily |

**18d. On days that you inhale or smoke cannabis, how many times per day do you use it?** *[Please allow a drop down option so respondents can choose the specific number of times they use cannabis per day]*

|__|__|

**18e. How long have you inhaled or smoked cannabis?**

|__|__| months *or* |__|__| years

**19a. When you eat or drink cannabis products, what do you consume?** (Check all that apply)

🞏 I eat baked goods I buy at the store

🞏 I eat homemade baked goods

🞏 I eat candy/other edible items

🞏 I eat butter or oils

🞏 I drink beverages

🞏 I consume drops or tinctures (e.g., under my tongue or combined with foods I cook at home)

🞏 I consume capsules or pills

**19b. The State of Ohio considers "CBD products" to contain either no or almost no (not more than 0.3%) THC, the ingredient in cannabis that can cause a euphoria or a "high". Some CBD products have no THC at all. Products with higher amounts of THC are considered to be "marijuana products".**

**What type of cannabis product do you eat or drink? (Select one)**

🞏 No, I *only* consume THC-free cannabis products

🞏 Yes, I *only* consume cannabis products that contain THC

🞏 I consume some products that contain THC and some that do not

🞏 I don’t know

**19.b.i. Each time you use CBD products what dose do you typically use? [If selected 1 or 3 in 19b.]**

** ____ mg (or mg/ml)**

** Other: ________**

** I don’t know**

**19c. How often do you eat or drink cannabis products?** (Select one)

| 🞏 | 🞏 | 🞏 | 🞏 | 🞏 | 🞏 | 🞏 | 🞏 |
| --- | --- | --- | --- | --- | --- | --- | --- |
| Less than once per month | 1 day per month | 2-3 days per month | 1 day per week | 2 days per week | 3-4 days per week | 5-6 days per week | Daily |

**19d. On days that you eat or drink cannabis products, how many times per day do you use it?** *[Please allow a drop down option so respondents can choose the specific number of times they use cannabis per day]*

|__|__|

**19e. How long have you eaten or drank cannabis products?**

|__|__| months *or* |__|__| years

**20a. When you apply cannabis products to your skin, what do you use?** (Check all that apply)

🞏 I use skin patches (“transdermal patch”)

🞏 I use lotions, balms, creams, or ointments

**20b.** **The State of Ohio considers "CBD products" to contain either no or almost no (not more than 0.3%) THC, the ingredient in cannabis that can cause a euphoria or a "high". Some CBD products have no THC at all. Products with higher amounts of THC are considered to be "marijuana products".**

**What type of cannabis product do you apply to your skin? (Select one)**

🞏 No, I *only* apply THC-free cannabis products to my skin

🞏 Yes, I *only* apply cannabis products that contain THC to my skin

🞏 I apply some products that contain THC and some that do not to my skin

🞏 I don’t know

**20.b.i. Each time you use CBD products what dose do you typically use? [If selected 1 or 3 in 20b.]**

** ____ mg (or mg/ml)**

** Other: ________**

** I don’t know**

**20c. How often do you apply cannabis products to your skin?** (Select one)

| 🞏 | 🞏 | 🞏 | 🞏 | 🞏 | 🞏 | 🞏 | 🞏 |
| --- | --- | --- | --- | --- | --- | --- | --- |
| Less than once per month | 1 day per month | 2-3 days per month | 1 day per week | 2 days per week | 3-4 days per week | 5-6 days per week | Daily |

**20d. On days that you apply cannabis products to your skin, how many times per day do you do it?** *[Please allow a drop down option where respondents can choose the specific number of times they use cannabis per day]*

|__|__|

**20e. How long have you applied cannabis products to your skin?**

|__|__| months *or* |__|__| years

**21a. The State of Ohio considers "CBD products" to contain either no or almost no (not more than 0.3%) THC, the ingredient in cannabis that can cause a euphoria or a "high". Some CBD products have no THC at all. Products with higher amounts of THC are considered to be "marijuana products".**

**What type of other cannabis product do you use? (Select one)**

🞏 No, I *only* use THC-free OTHER cannabis products

🞏 Yes, I *only* use OTHER cannabis products that contain THC

🞏 I use some products that contain THC and some that do not

🞏 I don’t know

**21.a.i. Each time you use CBD products what dose do you typically use? [If selected 1 or 3 in 21a.]**

 ____ mg (or mg/ml)

 Other: ________

 I don’t know

**21b. How often do you use OTHER cannabis products?** (Select one)

| 🞏 | 🞏 | 🞏 | 🞏 | 🞏 | 🞏 | 🞏 | 🞏 |
| --- | --- | --- | --- | --- | --- | --- | --- |
| Less than once per month | 1 day per month | 2-3 days per month | 1 day per week | 2 days per week | 3-4 days per week | 5-6 days per week | Daily |

**21c. On days that you use OTHER cannabis products, how many times per day do you do it?** *[Please allow a drop down option so respondents can choose the specific number of times they use cannabis per day]*

|__|__|

**21d. How long have you used OTHER cannabis products?**

|__|__| months *or* |__|__| years

**22a. Why are you using cannabis currently?** (Check all that apply) *[For each selected response: branch to 22b so that each response has option to select symptom improvement]*

🞏 Depression/to improve mood 🞏 To improve my appetite

🞏 To improve my pain 🞏 To treat my cancer

🞏 To improve my nausea/stomach upset 🞏 To help me cope with my illness

🞏 I use cannabis recreationally/for enjoyment 🞏 To help me deal with stress

🞏 To help me sleep

🞏 Other reason (describe):__________________________________________________________________

**22b. To what degree has cannabis helped to improve your symptom(s)?** (Select one)

1 -------- 2 -------- 3 -------- 4 -------- 5 ---------- 6 -------- 7 -------- 8 -------- 9 -------- 10

Minimal Moderate Major

**23. Do you feel like you want to stop using cannabis?**

🞏 YES 🞏 NO

**24. Are you aware of any potential health risks associated with cannabis use during your cancer treatment?** *[Allow responses to be open ended for participants to type into text field]*

🞏 NO 🞏 YES – Which risks? Please describe: ___________________________________________

**25. Did your doctor or another healthcare provider discuss any of the potential health risks associated with using marijuana during cancer treatment?** *[Allow responses to be open ended for participants to type into text field]*

🞏 NO 🞏 YES – Which risks? Please describe: ___________________________________________

**26. Do you feel comfortable discussing marijuana with your cancer doctor?**

🞏 YES 🞏 NO

**27. Do you feel comfortable discussing marijuana with your primary care doctor?**

🞏 YES 🞏 NO 🞏 Don’t have a primary care doctor

**BACKGROUND INFORMATION**

**28. Age:** |__|__|years

**29. Gender:** 🞏 Male 🞏 Female 🞏 Non-binary/third gender 🞏Prefer not to say

**30. Which of the following best describes your racial or ethnic background:** (Check all that apply)

🞏 White

🞏 Hispanic

🞏 Black or African-American

🞏 American Indian or Alaska Native

🞏 East Asian, Southeast Asian, or Pacific Islander

🞏 South Asian

🞏 Other

1. **What state do you live in?** *[DROP DOWN US STATES w/Ohio and bordering states first]*
2. **What county do you live in?** *[DROP DOWN COUNTIES for Ohio only]*
3. ***What is your zip code of residence?*** |__|__|__|__|__|
4. **What is the highest level of school that you have completed?**

🞏 Grade school or some high school

🞏 High school graduate or G.E.D.

🞏 Some college, technical school graduate, or Associates degree

🞏 Bachelor’s degree/4 year college graduate

🞏 Graduate degree (e.g. Master’s, JD, PhD)

1. **Presently, has your cancer spread?**

🞏 No *[Go to 34]*

🞏 Yes *[Continue to 33a]*

🞏 I don’t know *[Go to 34]*

**35a. How has your cancer spread?** (Select one)

🞏 My cancer has spread locally/regionally to areas close to the tumor’s original location (e.g. lymph nodes)

🞏 My cancer has spread far away to other parts of my body (e.g. brain, liver, bone)

🞏 My cancer has spread but I don’t know how much or how far

1. **Where are you in your cancer treatment?**

🞏 I am currently undergoing treatment *[Go to 35]*

🞏 I have finished treatment/surgery and am here for follow-up/check-up *[Go to 35]*

🞏 I have not yet received treatment/surgery *[End of survey]*

🞏 I do not plan to receive treatment or surgery *[End of survey]*

**37. Please indicate if you have completed, are currently receiving, or are planning to receive any of the following cancer treatments:**

**37a. Radiation therapy** *(Radiation therapy is a type of cancer treatment that uses high doses of radiation to kill cancer cells and shrink tumors. Radiation therapy is used to treat cancer and to reduce cancer symptoms).*

(Select one)

🞏 I completed radiation therapy for my cancer *[Go to 35a1]*

🞏 I am currently receiving radiation therapy for my cancer *[Go to 35b]*

🞏 I am planning to receive radiation therapy for my cancer *[Go to 35b]*

🞏 Radiation therapy is not part of the treatment plan for my cancer *[Go to 35b]*

🞏 I’m not sure *[Go to 35b]*

**37b. Chemotherapy** *(Chemotherapy or “chemo” is a type of medicine taken as a pill or intravenously (“I.V.”). These drugs are intended to cure, control, or offer pain relief for cancer patients).* (Select one)

🞏 I completed chemotherapy for my cancer *[Go to 35b1]*

🞏 I am currently receiving chemotherapy for my cancer *[Go to 35c]*

🞏 I am planning to receive chemotherapy for my cancer *[Go to 35c]*

🞏 Chemotherapy is not part of the treatment plan for my cancer *[Go to 35c]*

🞏 I’m not sure *[Go to 35c]*

**37c. Immunotherapy** *(Immunotherapy is a type of cancer treatment that helps your immune system fight cancer. The immune system helps your body fight infections and other diseases).* (Select one)

🞏 I completed immunotherapy for my cancer *[Go to 35c1]*

🞏 I am currently receiving immunotherapy for my cancer *[Go to 35d]*

🞏 I am planning to receive immunotherapy for my cancer *[Go to 35d]*

🞏 Immunotherapy is not part of the treatment plan for my cancer *[Go to 35d]*

🞏 I’m not sure *[Go to 35d]*

**37d. Bone marrow transplant** *(A bone marrow or stem cell transplant is a treatment for some types of cancer that replaces damaged cells with healthy ones from your own or a donor’s bone marrow. For example, you might have one if you have leukemia, multiple myeloma, or some types of lymphoma).* (Select one)

🞏 I had a bone marrow transplant for my cancer *[Go to 35d1]*

🞏 I am currently receiving a bone marrow transplant for my cancer *[Go to 35e]*

🞏 I am planning to receive a bone marrow transplant for my cancer *[Go to 35e]*

🞏 A bone marrow transplant is not part of the treatment plan for my cancer *[Go to 35e]*

🞏 I’m not sure *[Go to 35e]*

**37e. Surgery** *(Surgery is a procedure in which a doctor remove cancer from your body).* (Select one)

🞏 I completed surgery for my cancer *[Go to 35e1]*

🞏 I am planning to undergo surgery for my cancer

🞏 Surgery is not part of the treatment plan for my cancer

🞏 I’m not sure

**TOBACCO INFORMATION**

1. **Have you ever smoked cigarettes fairly regularly?**

🞏 YES *[Continue to 38]* 🞏 NO *[Go to 39]*

1. **Do you smoke cigarettes now?**

🞏 YES 🞏 NO *[Go to 38a]*

**39a**. **How long ago did you stop smoking cigarettes regularly?**

🞏 More than 10 years ago 🞏 Less than 10 years ago

**The next questions are about electronic cigarettes, often called e-cigarettes, vape pens, or mods. E-cigarettes re battery-powered and produce vapor instead of smoke.**

1. **Aside from cannabis products, have you ever used electronic cigarettes (vaping devices) fairly regularly?**

🞏 YES *[Continue to 42]* 🞏 NO *[Go to 46]*

1. **Do you smoke e-cigarettes now?**

🞏 YES *[Continue to 43]* 🞏 NO *[Go to 42a]*

**OPIOID INFORMATION**

The next questions are about opioids. Opioids are a type of medication used for pain relief. Opioids are a type of medication used for pain relief. Some common examples include Oxycodone (Oxycotin), hydrocodone (Vicodin), morphine, fentanyl, methadone, tramadol, codeine and Percocet.

1. **Do you currently use opioids for pain relief?**

🞏 No *[Go to 46]*

🞏 Yes *[Continue to 43]*

🞏 Im taking a pain medication but I don’t know if it’s an opioid) (please tell us the name if possible) *[continue 43]*

🞏 I don’t know *[go to 46]*

1. **How often do you use opioid medications?**

| 🞏 | 🞏 | 🞏 | 🞏 | 🞏 | 🞏 | 🞏 | 🞏 |
| --- | --- | --- | --- | --- | --- | --- | --- |
| Less than once per month | 1 day per month | 2-3 days per month | 1 day per week | 2 days per week | 3-4 days per week | 5-6 days per week | Daily |

1. **Do you use opioid medications for cancer-related pain?**

🞏 No *[Go to 46]*

🞏 Yes *[Continue to 45]*

1. **How well do your opioids relieve your cancer-related pain?**

1 -------- 2 -------- 3 -------- 4 -------- 5 ---------- 6 -------- 7 -------- 8 -------- 9 -------- 10

Minimal Moderate Major

**BENZODIAZAPINE INFORMATION**

The next questions are about benzodiazepines and similar medications. Benzodiazepines and similar medications are a type of medication commonly used for anxiety and sleep.

Some common examples include Atavan (Lorazepam), Xanax (Alprazolam), Klonopin (Clonazepam), Ambien (Zolpidem). Do you CURRENTLY use benzodiazepines or similar medications to relieve anxiety or help you sleep?

1. **Do you use currently use benzodiazepines or similar medication to relive anxiety or help you sleep?**

🞏 No *[end of survey]*

🞏 Yes *[Continue to 47]*

1. **How often do you use these drugs?**

| 🞏 | 🞏 | 🞏 | 🞏 | 🞏 | 🞏 | 🞏 | 🞏 |
| --- | --- | --- | --- | --- | --- | --- | --- |
| Less than once per month | 1 day per month | 2-3 days per month | 1 day per week | 2 days per week | 3-4 days per week | 5-6 days per week | Daily |

1. **Do you use benzodiazepine medication for reasons related to your cancer diagnosis?**

🞏 No *[end of survey]*

🞏 Yes *[Continue to 49]*

1. **How well do these medications help you sleep or feel less anxious?**

1 -------- 2 -------- 3 -------- 4 -------- 5 ---------- 6 -------- 7 -------- 8 -------- 9 -------- 10

Minimal Moderate Major

[end of survey]
